# Pond chironomid communities revealed by molecular species delimitation reflect eutrophication

**DOI:** 10.1002/ece3.7315

**Published:** 2021-03-23

**Authors:** Kenzi Takamura, Ryuhei Ueno, Natsuko Ito Kondo, Kako Ohbayashi

**Affiliations:** ^1^ Center for Environmental Biology and Ecosystem National Institute for Environmental Studies Tsukuba Japan; ^2^ Graduate School of Arts and Sciences The University of Tokyo Komaba Japan

**Keywords:** chironomid community, coalescence, DNA barcoding, farm pond, freshwater biodiversity, land use

## Abstract

Farm ponds, a valued habitat for freshwater organisms, are being negatively affected by the recent changes in the environment as well as anthropological activities. In these ponds, biodiversity researchers have tended to focus on species that prefer natural habitats and/or can be identified based on morphological characters. In contrast, this study focused on the insect family Chironomidae, which is widely distributed from clear to polluted waters of ponds, but is hard to identify morphologically as an aquatic larva. We adopted DNA barcoding and molecular species delimitation to identify every single specimen of quantitative collections. From bottom sediments of 17 ponds in summer in the Banshu Plain of Japan, a total of 62 species were delimited based on the DNA sequences of the mitochondrial COI region. Chironomid communities from these ponds were classified into four groups in a two‐dimensional ordination of multivariate analysis (NMDS). One of the dimensions was well correlated with the gradient of eutrophication, while another dimension was not clearly assigned to any general feature of the environmental gradient, but rice cultivation could possibly be involved.

## Graphical abstract

This study focused on the insect family Chironomidae in farm ponds with assistance of DNA barcoding and molecular species delimitation. Chironomid communities were classified into four groups in a NMDS analysis. One dimension is associated with eutrophication and another possibly with rice culture.

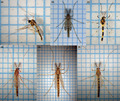

## 1 INTRODUCTION

Farm ponds in Japan originally constructed for agricultural irrigation are now at the brink of disappearance not only due to shrinking local agricultural production, but also in order to prevent flooding of the ponds due to abnormally heavy rainfall. They are, however, habitats for endemic aquatic flora and fauna, and thus sources and refuges for aquatic organisms that can also be found in lakes, rivers, and wetlands (Takamura, 2012). They are highly valued in terms of conserving freshwater biodiversity even with the on‐going changes. The structure, size, water inflow and outflow, influx of inorganic and organic substances as well as the surrounding landscapes of the ponds have given rise to a wide variety of pond environments. These respective environments are habitats that actually or potentially nurture a unique set of aquatic flora and fauna (Casas et al., 2012; Cereghino et al., 2008; Chester & Robson, 2013; Fuentes‐Rodriguez et al., 2013; Takamura, 2012).

A large number of farm ponds, most of which are/were small‐scale, have been created in a seasonally dry temperate climate, such as the coastal area of the Setonaikai Sea (hereafter the Setonaikai Coast) in Japan. Researchers have focused on their value as a habitat of biodiversity in Japan as well as in other areas with such ponds (Casas et al., 2012). They have focused on the study of vascular plants, insects like odonates, hemipterans, and beetles, and vertebrates like fishes, annulans, and frogs (Fukumori et al., 2016; Iwai et al., 2017; Natsumeda et al., 2015; Usio et al., 2017). In such studies, most of the taxa studied are sensitive to environmental deterioration, now in progress among farm ponds, and thus they can be indicators of well‐conserved waters. However, these species have been disappearing in highly deteriorated waters and indicate the deterioration by their absence (Ito et al., 2020). There are other taxa containing species that can inhabit those waters and contribute to ecosystem function through their presence. Including them in studies of pond ecosystems would provide a more detailed and vivid picture of pond biodiversity.

Adding to this, those taxa widely focused on in studies of pond biodiversity have often been ones for which their taxonomy is accessible through morphological identification. However, there are quite a few taxa which are not easily identified that abundantly appear in farm ponds. They are also an indispensable part of the pond biodiversity and may play a role in the ecosystem inside and outside the pond. One excellent way of revealing their taxonomic entity is the molecular identification of those organisms, that is, DNA barcoding (Hebert et al., 2003, 2016). This method of research will enhance recognition of the value of biodiversity in ponds and that of the ponds themselves in the arena of the freshwater biodiversity landscape.

**FIGURE 1:**
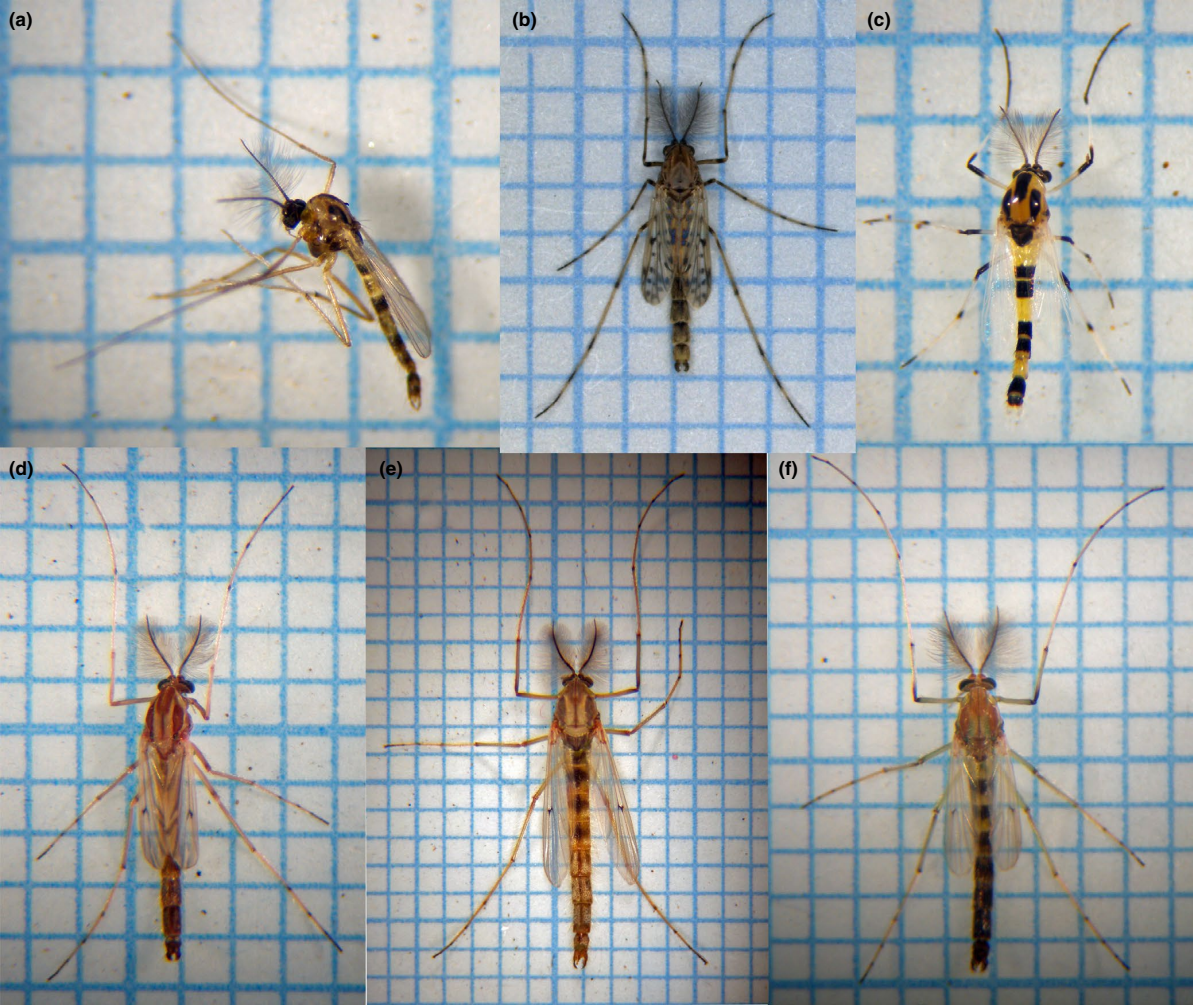
Adult males of chironomids. They were collected on the shores of the study ponds and identified to species morphologically, but not on DNA barcodes. (a) *Polypedilum tigrinum* (collected at Minamishin Pond), (b)*Tanypus* species (Jo Pond), (c)*Cricotopus trifasciatus* (Shinju Pond), (d) *Chironomus kiiensis* or *C. striatipennis* (Sobuchi Pond), (e) *Chironomus plumosus* (Jo Pond), (f) *Chironomus fujitertius* (Shinju Pond)

Integrating these two points together, chironomids are one of the representative taxa that should be studied among a wide range of pond environments. A large number of chironomid species are observed in ponds (Figure 1). They are one of the major groups of animals that occur in freshwater habitats like farm ponds, paddy fields, rivers, and lakes (Casas & Langton, 2008; Fuentes‐Rodriguez et al., 2013; Medeiros & Quinlan, 2011). The species composition of chironomids varies drastically between different waters, so they have been regarded as good indicator organisms for freshwater environments (Lindegaard, 1995). For example, an index was introduced for a variety of lakes based on the composition of indicator chironomid species collected from the lakes, reflecting the level of eutrophication observed there (Wiederholm, 1980).

Aquatic chironomid larvae are found throughout the seasons in freshwaters that have a wide variety of water quality and substrate composition and can be collected with simple gear like an Ekman‐Birge grab sampler in lakes and ponds or a Surber net in rivers and streams. So, they are good materials for investigating the actual state of the freshwater environment. But one of the difficulties of understanding the chironomid fauna in the water is identifying all chironomid species as larvae correctly from their morphologies. Based on the morphological keys and diagnoses that have been developed worldwide or for the specific local area (e.g., Andersen et al., 2013; Japan Chironomid Workshop, 2010), a number of chironomid species can be identified as larvae, but most species are hard to identify. Morphological features the larva have are often not taxonomically informative or require painstaking measure/high magnification and expertise to distinguish. However, recent developments in DNA barcoding (Hebert et al., 2003, 2016) have offered a more objective and effective way to perceive the taxonomy of morphological character‐poor creatures like larval chironomids, given a wealth of DNA barcodes (species‐specific DNA sequences) for identifying biological species on a molecular basis.

DNA barcoding studies have been made for chironomids in several areas (e.g., Brodin et al., 2013; Ekrem et al., 2010; Kondo et al., 2016; Pfenninger et al., 2007; Sinclair & Gresens, 2008). A similar project is also being undertaken in Japan and has come to fruition as the Chironomid DNA Barcode Database (National Institute for Environmental Studies: http://www.nies.go.jp/yusurika/).

As stated before, among farm ponds densely spaced on the Banshu Plain of the Setonaikai Coast, biodiversity research has been intensively undertaken since the 1990s (Takamura, 2012). They are based on species identification of organisms collected or observed in and around ponds with the assistance of taxonomical specialists proficient in morphological identification. However, many taxa that are not easily identified by morphological taxonomy have remained untouched in these researches. Researches on chironomids of bottom sediments and aquatic weeds in summer and fall, 2012 were made in these ponds based on the molecular species delimitation of larval chironomids, and only the species number of chironomids was compared between ponds and with other studies in Takamura et al., (2017). This report focuses on the effects of water quality, surrounding land use, and aquatic flora on the benthic chironomid communities of bottom sediments in the summer. Chironomid communities identified from the farm ponds were classified into several groups and related to a certain set of environmental conditions. The results and DNA barcodes gained during the course of DNA barcoding are reported here.

## 2 MATERIALS AND METHODS

### 2.1 Study ponds

**FIGURE 2:**
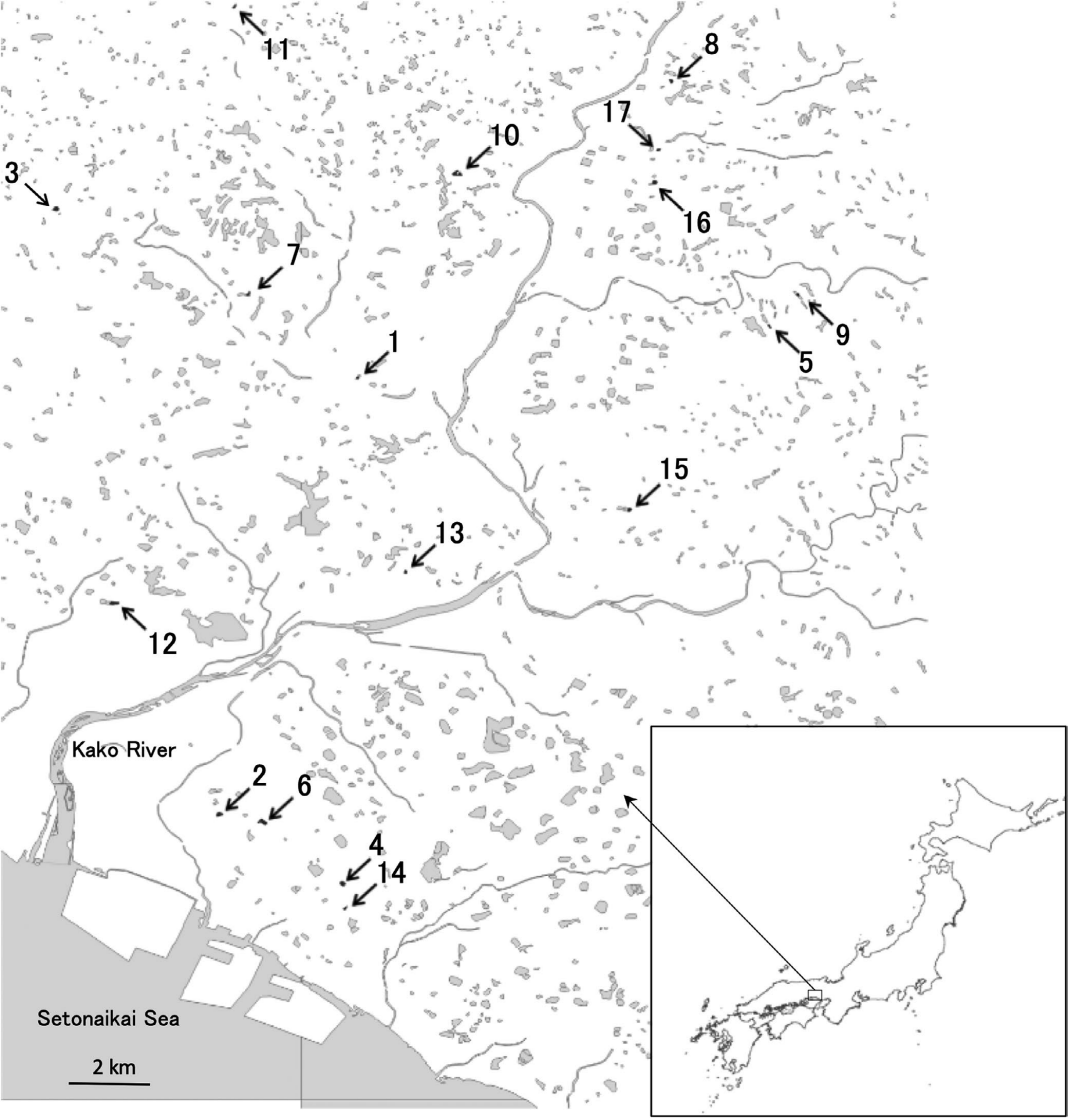
Locations of the study ponds on the Banshu Plain. The ponds are outlined with thick lines. Pond numbers are the same as in Table 1

Twenty study ponds were selected for sampling chironomids from among farm ponds in the eastern part of the Banshu Plain (Takamura et al., 2017) to compare chironomid communities from different environmental conditions in the same year. Of these ponds, 17 ponds were ultimately included in this study (Figure 2), while the other three ponds were omitted due to the poor collection of chironomids. Their landscape surroundings were regarded as six arable, six forest, and five urban ponds (Table 1), but all of the locations were in the Satoyama (the diverse mosaic of agricultural and nonagricultural lands: Kadoya & Washitani, 2011; Washitani, 2001) area and contained all of the three elements to various degrees. They were small, up to 2 ha in area, and as deep as 6 m in maximum depth. They could be separated into two types. One was the excavated type, which was formed with natural or artificial excavation on a relatively flat landscape, and the other was the embankment type, which was built by embanking a valley.

**TABLE 1:** List of study ponds with the grouping on NMDS ordination and 18 environmental variables

No.	Pond name	Landscape	Type	Group	Dpth	IL	BGAr	chla	DO	Secci	spcond	SS	TN	TP	wplantR	bank	Trapa	City	Water	Rice	Woods	Field
1	Abikishimo	Arable	Exc	D	0.93	0.277	0.7	6.0	9.4	1.00	0.184	6.24	1.753	0.022	1.5	0.0	0	0.000	0.241	0.688	0.000	0.071
2	Ashi	Urban	Exc	C	0.70	0.140	2.7	20.4	3.1	0.19	0.226	22.77	1.013	0.269	3.75	1.0	0	0.445	0.003	0.552	0.000	0.000
3	Fukuzumishin	Forest	Emb	B	2.73	0.048	0.5	6.5	7.3	1.39	0.050	8.91	0.522	0.031	2.5	0.0	0	0.000	0.062	0.168	0.770	0.000
4	Jo	Urban	Exc	C	0.61	0.106	40	78.8	13	0.35	0.215	43.50	2.530	0.348	0	0.7	0	0.605	0.058	0.337	0.000	0.000
5	Kashitanirokugo	Forest	Emb	A	4.68	0.059	0.2	3.2	8.7	1.99	0.041	2.85	0.284	0.020	1	0.2	0	0.000	0.105	0.000	0.895	0.000
6	Kazu	Urban	Exc	C	0.79	0.310	11	71.5	13	0.66	0.173	16.72	1.565	0.131	0	0.7	0	0.978	0.022	0.000	0.000	0.000
7	Kita‐Tanaka	Arable	Emb	B	2.22	0.088	10	44.0	16	0.76	0.239	8.64	2.132	0.305	0	0.2	0	0.000	0.238	0.000	0.000	0.762
8	Konpira	Arable	Exc	D	0.78	0.032	0.4	2.9	9.9	0.68	0.050	10.80	0.341	0.041	1.5	0.0	0	0.110	0.048	0.841	0.001	0.000
9	Kuriyanigo	Forest	Emb	A	3.92	0.048	0.1	2.2	8.8	2.86	0.079	1.63	0.217	0.010	0	0.3	0	0.050	0.302	0.043	0.606	0.000
10	Minamishin	Arable	Exc	D	0.63	0.063	1.9	69.7	5	0.94	0.064	18.23	0.959	0.093	2.5	0.8	1	0.496	0.109	0.395	0.000	0.000
11	Hiroharamuko	Forest	Emb	A	3.15	0.072	1.6	11.6	9.6	1.75	0.043	4.68	0.349	0.033	0.5	0.0	1	0.012	0.079	0.549	0.360	0.000
12	Shinhitsu	Arable	Exc	D	1.20	0.045	5.9	22.2	12	0.41	0.177	22.65	1.091	0.356	0.5	0.8	0	0.000	0.329	0.554	0.000	0.117
13	Shinju	Arable	Emb	D	1.07	0.055	1.9	33.5	7.8	0.44	0.142	33.26	0.648	0.115	1	0.2	1	0.000	0.072	0.134	0.338	0.455
14	Sobuchi	Urban	Exc	C	0.71	0.035	1.7	56.2	2	0.33	0.119	21.93	1.457	0.139	2.75	1.0	1	0.943	0.057	0.000	0.000	0.000
15	Tosaka	Forest	Emb	A	2.63	0.044	0.4	5.7	9.9	1.77	0.037	5.84	0.287	0.033	1	0.4	0	0.000	0.302	0.106	0.593	0.000
16	Yashiro‐o	Arable	Exc	B	0.87	0.088	20	85.0	14	0.35	0.120	39.30	1.609	0.236	0	0.7	0	0.526	0.146	0.328	0.000	0.000
17	Ieharakami	Urban	Emb	B	3.24	0.069	1.4	14.8	9.1	1.43	0.134	4.27	0.544	0.059	0.75	0.8	1	0.971	0.029	0.000	0.000	0.000

Note

Pond types are excavated (Exc) and embanked (Emb). Dpth: maximum bottom depth of the pond (m), IL: organic content of the bottom substrate as ignition loss, BGAr: phycocyanin concentration of water (RFU: relative fluorescent unit), chla: chlorophyll a concentration of water (µg/l), DO: dissolved oxygen (mg/l), Secci: water transparency (m), spcond: specific conductivity (mS/cm), SS: suspended solid (mg/l), TN: total nitrogen (mg/l), TP: total phosphorus (mg/l), wplantR: sum of the cover scores for water plant, bank: ratio of pond perimeter covered with artificial bank, Trapa: cover score for *Trapa japonica*, city, water, rice, woods, and field: ratios of area covered with urban area, open water, rice paddy, forest, and agricultural field except for rice paddy.

### 2.2 Chironomid collection

The original sample collection was conducted from bottom sediments and aquatic weeds in May and September 2012 (Takamura et al., 2017). For benthic chironomids, a sampling researcher approached the central part of the pond on a fishing floater and took three cores of bottom sediment with an Ekman‐Birge grab sampler (15 cm square in the mouth) in each pond. The sediment cores were sieved on the water with a NGG40 nylon mesh (470 µm opening) bag. The remains were kept in polyethylene bags and brought back to the laboratory in coolers on ice. The chironomids were sorted on ice in order to restrain degradation of DNA and preserved individually in 1.5 ml plastic tubes in a freezer for DNA analysis. Morphological identification was performed during the sorting based on the morphological keys (e.g., Andersen et al., 2013; Japan Chironomid Workshop, 2010), and a small number of larvae with unique morphological characters were identified as species.

### 2.3 DNA extraction and sequencing

Chironomid DNA was extracted in lysis buffer (1 mM Tris‐HCl (pH 8.0), 1 mM EDTA (pH 8.0), and 25 mM NaCl) containing proteinase K during the incubation for 16–18 hr at 56°C. The extracted DNA was amplified for the mitochondrial COI gene with the standard primer set (Folmer et al., 1994) and KOD Fx Neo polymerase (Toyobo Co.) with the supplementary buffer. The PCR reaction was an initial step of 95°C for 2 min, followed by 40 cycles of 98°C for 10 s, 51°C for 30 s and 68°C for 1 min. The PCR products were purified using ExoSAP‐IT (Affymetrix) and subjected to direct sequencing using a BigDye DNA Terminator v3.1 Cycle Sequencing Kit (Life Technologies) and an ABI 3730 Genetic Analyzer (Applied Biosystems). In the previous report (Takamura et al., 2017; May and September 2012), we collected 1,060 larval or pupal chironomids from bottom sediments and aquatic weeds, of which 786 individuals were successfully sequenced. In this report, our samples were 443 larvae from the bottom sediments in May 2012, of which 296 individuals were successfully sequenced. The percentages of successfully sequenced individuals were higher than 50% in most of the ponds, but they were as low as 9% and 25% in Minamishin and Hiroharamukou, respectively. The low percentages were probably due to imperfect icing conditions during the sample transportation after collection or humic substances derived from plant debris collected with bottom sediments (Wnuk et al., 2020). The accession numbers of the DNA sequences obtained in all 20 ponds are LC494722–LC495141 and those referred to in this study are listed in Table S1.

### 2.4 Species delimitation based on DNA sequences

**FIGURE 3:**
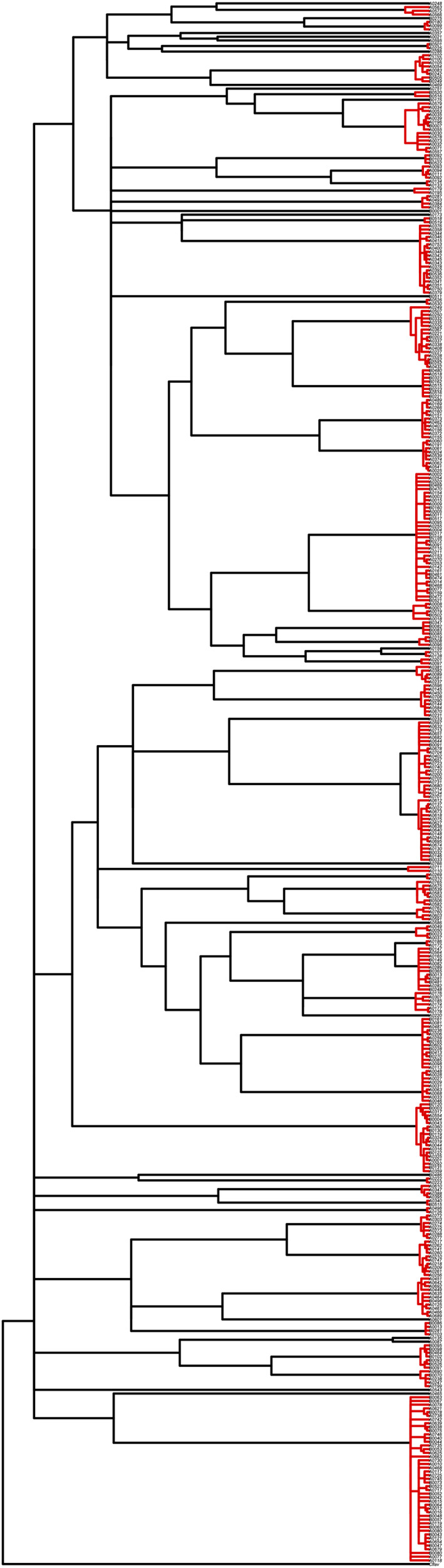
Species delimitation results by GMYC on a strict‐clock tree reconstructed by MrBayes software. A cluster of branches continuously colored in red represents a delimited species (molecular operational taxonomic unit: MOTU). A singleton also represents a single species. Each haplotype sequence is specified with the specimen ID of each sample individual. Specimen IDs and species names are in Table S3. Node reliabilities are omitted

**FIGURE 4:**
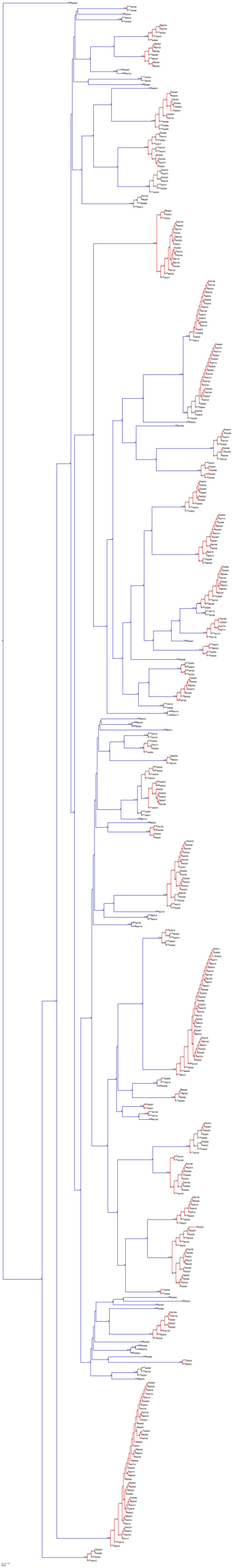
Species delimitation results by PTP on a nonclock tree reconstructed by MrBayes software. A cluster of branches continuously colored in red represents a delimited species (molecular operational taxonomic unit: MOTU). A singleton also represents a single species. Each haplotype sequence is specified with the specimen ID of each sample individual. Specimen IDs and species names are in Table S4

The COI DNA sequences of the chironomid specimens were collapsed to haplotypes on the FaBox platform (Villesen, 2007). The haplotype sequences were aligned and read by MrBayes software 3.2.0 (Huelsenbeck & Ronquist, 2001; Ronquist & Huelsenbeck, 2003) to reconstruct a phylogenetic tree under a molecular clock model (strict clock) and a nonclock model. The best model of nucleotide substitution was selected as GTR + I + G with the jModelTest 2 (Darriba et al., 2012; Guindon & Gascuel, 2003). Species were delimited on the phylogenetic trees with different algorithmic methods. Species delimitation was made on the molecular clock tree with General Mixed Yule Coalescence (GMYC: Fujisawa & Barraclough, 2013; Pons et al., 2006). The single threshold level (Fujisawa & Barraclough, 2013) was adopted to segregate the speciation and the coalescence bifurcation on the tree. It was also made on the nonclock tree with Poisson Tree Process (Zhang et al., 2013), which was performed on the bPTP web server (http://species.h‐its.org/ptp/) setting the number of MCMC generations as 500,000. Species delimited by these methods were 72 species for GMYC and 80 species for PTP in May and September for bottom sediments and aquatic weeds (Takamura et al., 2017; Tables S3 and S4, Figures 3 and 4). They were compared and lumped in the way that the resulting species number was the lowest. For each species unit delimited, scientific names were determined by a BLAST search on the website of the DNA Data Bank of Japan (DDBJ: https://www.ddbj.nig.ac.jp/services‐e.html) with ≥97% identity. Species found from bottom sediments in May 2012 were listed for this study (Table S1).

### 2.5 Ordination of chironomid communities

Chironomid communities of the 17 farm ponds were classified by nonmetric multi‐dimensional scaling (NMDS) using the R program package vegan (Oksanen, 2015) with the application of the similarity index of Chao et al. (2006). Indicator species were screened for the classified groups using the package labdsv (Roberts, 2016).

A total of 18 environmental variables to be related to the ordination were obtained from measurements of the aquatic physical, chemical and biological environments, the local government statistics on pond status, and the government map of land use and vegetation. The original land‐use and vegetation maps (each 10 km square on the 1/25,000 scale) were obtained from the Japan Integrated Biodiversity Information System (J‐IBIS; Ministry of the Environment) and compiled by Akasaka et al. (2010). We exchanged the unit maps when J‐IBIS renewed them after the compilation by Akasaka et al. (2010). Land use ratios were obtained within the 50‐m buffer zone from the pond perimeter on the map using ArcGIS Desktop ver. 10.2 (ESRI). The land use limited to the vicinity of the ponds was adopted because the visual criteria for choosing the study ponds fitted for this range of landscape, although different landscape ranges might have affected the pond environments as well as the chironomid communities.

Aquatic environmental variables, which are expected to affect chironomid communities, were selected from measurements of a pond water‐quality study (Kizuka, unpublished). Water transparency (Secci), dissolved oxygen (DO), suspended solids (SS), specific conductivity (spcond), chlorophyll a (chla), and phycocyanin concentration as a substitute for cyanobacteria abundance (BGAr) were measured on‐site with a Secci disk, a DO meter model 58 (YSI) or a multiparameter water quality sonde model 6600‐D (YSI) during the daytime, and total phosphorus (TP) and nitrogen (TN) concentrations were measured from collected pond water. These measurements were made once each month from May to September 2012.

Coverage of all water plants (wplantR) or *Trapa japonica* (Trapa) was scored visually with the naked eye as 1 (75%–100% cover), 0.75 (50%–75%), 0.5 (25%–50%), and 0 (0%–25%) in August 2012. *T. japonica*, a floating‐leaf plant, often covered the entire pond surface and it is associated with poor DO and light on the bottom (Takamura et al., 2003). The ratio of pond perimeter covered with an artificial bank (mainly concrete) was measured in the same year. The organic content of the bottom substrate as ignition loss (IL) was measured with the samples of the surface (1–2 cm thick) muddy substrate collected in September 2012. The substrate was heated at 360 ˚C for 2 hr (Salehi et al., 2011).

All of the environmental variables (Table 1) were projected on the ordination as explanatory factors. Variations in all of the environmental variables were summarized with principal component analysis and also projected on the ordination as explanatory factors. The PCA analysis was performed with the "prcomp" function in the R. Land use ratios (forest (wood), urban area (city), ponds, rivers, and lakes (water), rice paddy (rice), and agricultural field except for rice paddy (field)), ignition loss as well as *T. japonica* cover and the ratio of artificial bank were logit‐transformed before the NMDS and PCA analyses. If the divided ratios were 1 and 0, they were replaced with 0.99 and 0.01, respectively, in the transformation.

## 3 RESULTS

**FIGURE 5:**
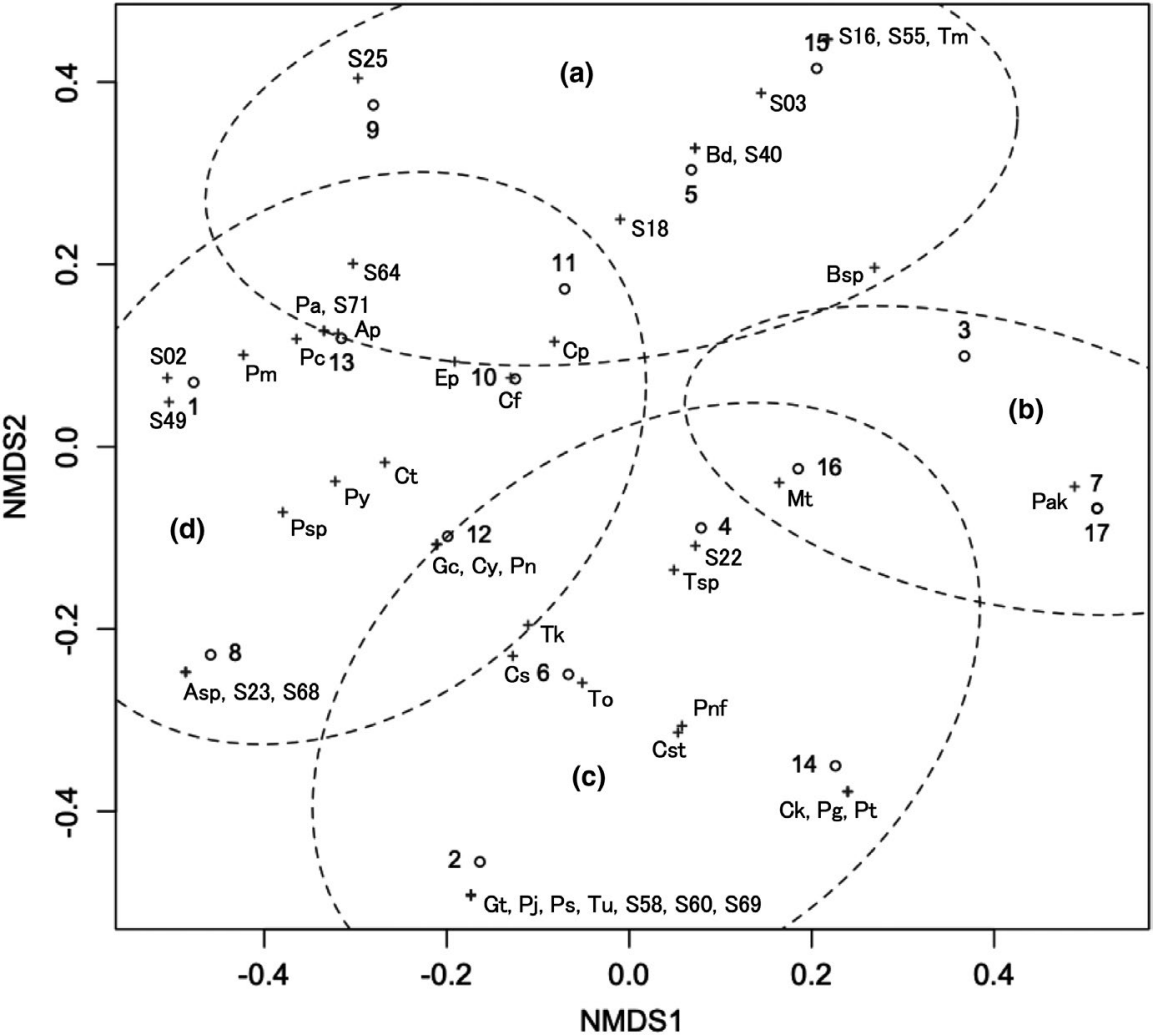
NMDS ordination plot of the ponds and chironomid species. The grouping of ponds is signified with a, b, c, d in paratheses and ellipses with broken lines showing the 90% confidence area. Circles: pond, crosses: chironomid species. Pond numbers and abbreviations of species names are the same as in Table 1 and Table S1, respectively

**FIGURE 6:**
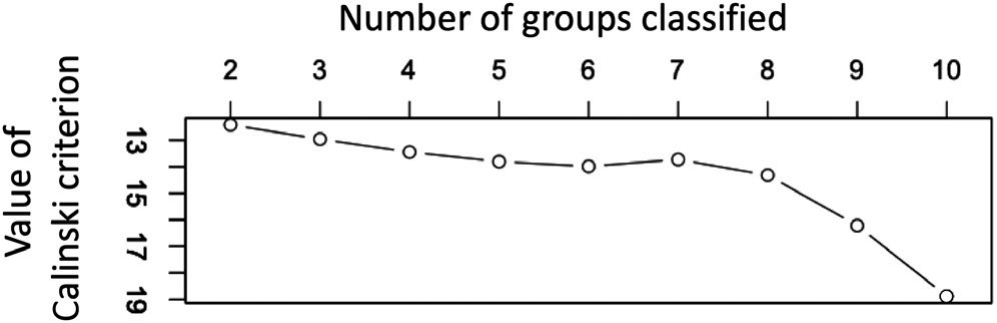
Plot of Calinski criterion for the groupings of pond chironomid communities. The vertical axis is inversely plotted

In all 17 farm ponds surveyed, 48 species were delimited from 177 haplotypes (Table S1). This species delimitation was based on 421 DNA haplotype sequences out of 786 chironomids from total 20 ponds (Takamura et al., 2017; Tables S3 and S4, Figures 3 and 4). The chironomid community compositions of 17 farm ponds (Table S2) were ordinated in a two‐dimensional space and classified into four groups: A, B, C, D (Table 1, Figure 5). The stress value was 0.1064, indicating the ordination result was reasonable (Zurr et al., 2007). The four‐group classification gave a slight, but visible, hump on the graph line in Figure 6 of the Calinski criterion and so was judged as the most likely (Calinski & Harabasz, 1974), although it was not much more so than the five‐group classification (Figure 6).

Group A was comprised of four ponds (Kashitanirokugo, Kuriyanigo, Hiroharamuko, and Tosaka). Eleven chironomid species were associated with this group, but no species was a definite indicator species. The most strongly associated was *Benthalia* sp. (Bsp, *p* =.10). Group B was comprised of four ponds (Fukuzumishin, Kita‐Tanaka, Yashiro‐o, and Ieharakami), and only *Propsilocerus akamusi* (Pak) was associated with this group as an indicator species (*p* =.002). Group C was comprised of four ponds (Ashi, Jo, Kazu, and Sobuchi), and 18 species were associated with this group. *Tanytarsus oyamai* (To) was regarded as an indicator species (*p* =.018), and *Tanypus kraatzi* (Tk) was the second‐most associated (*p* =.095). Ponds in this group were clumped in a relatively narrow area on the coastal plain, while ponds in the other three groups were scattered in the inland area of floodplains and river terraces (Figure 2). Group D was comprised of five ponds (Abikishimo, Konpira, Minamishin, Shinhitsu, and Shinju), and 18 species were associated. There was no indicator species, but Sp64 and *Psectrotanypus* sp. K1 (Psp) were two of the most associated (*p* =.092 and 0.096). The groups B and D were most different along the first axis of the ordination, and the groups A and C were so along the second axis.

**FIGURE 7:**
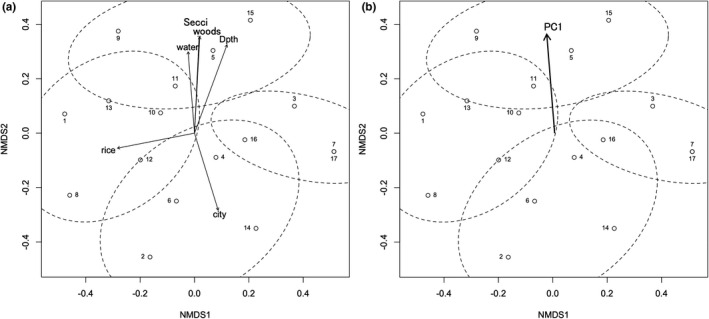
Environmental conditions of significance (*p* <.05) projected on the ordination plot of the pond chironomid communities. (a) Six of 18 environmental variables, (b) principal components of 18 environmental variables (only PC1 is projected). Abbreviations of environmental variables are the same as in Table 1

Of eighteen environmental variables projected on the ordination of the pond chironomid communities, water transparency (Secci), ratio of forest (woods), ratio of ponds, rivers, and lakes (water), depth (Dpth), ratio of rice paddy (rice), and ratio of urban area (city) had a significant correlation with the ordination (Figure 7a). The first four variables and the last one were in the opposite direction along the second axis of the ordination. This axis reflected a clear and turbid water gradient, indicating that the group C ponds were highly characterized by eutrophication, while the group A ponds were the least. Intuitively, the urban landscape of the former group and the forest landscape of the latter group were well reflected in the water quality. The groups B and D were intermediate on this axis. The ratio of rice paddy was largely independent of this gradient and nearly followed the first axis of the ordination.

The environmental variables were also summarized by the principal component analysis and projected on the ordination. Only the first principal component (PC1) showed a significant correlation with the ordination (Figure 7b). PC1 that accounted for 38.1% of the variation was represented by the following equation: PC1 = 0.302 Dpth − 0.170 IL − 0.280 BAGr − 0.312 chla − 0.090 DO + 0.326 Secci − 0.300 spcond − 0.306 SS − 0.331 TN − 0.314 TP + 0.027 wplantR − 0.208 bank − 0.008 Trapa − 0.200 city + 0.109 water − 0.034 rice + 0.308 woods − 0.076 field. This component likely represented a eutrophication gradient.

## 4 DISCUSSION

The present 17 pond chironomid communities were classified into four groups according to the compositions of delimited species. These results clearly show that there were different types of chironomid communities among the ponds which were different in the composition of surrounding land use. As this study is unique in adopting DNA barcoding and offering a well‐objective taxonomy of molecular species delimitation, it is worth mentioning what species is unique to the pond environments.

The second axis of the ordination was correlated with the eutrophication gradient. A similar gradient has been reported for the chironomid communities in lakes and ponds of the eastern Canadian Arctic (Medeiros & Quinlan, 2011). There was no definite indicator chironomid species of the clear water ponds (group A), but *Tanytarsus oyamai* was the indicator of the turbid water ponds (group C). This species is commonly found in Japanese rice paddy fields (Takamura, 1993, 1996; Takamura & Yasuno, 1986) and prefers water of high specific conductivity (>90 µS/cm: Kawai et al., 1998).

*Propsilocerus akamusi*, the indicator species of the group B ponds, is common to Japanese eutrophic lakes (Iwakuma, 1987, 1992; Takamura & Iwakuma, 1990; Yamagishi & Fukuhara, 1971), but this species did not characterize group C, the typical eutrophic‐water chironomid community. *P. akamusi* is notorious for breaking out in eutrophic lakes. Actually, this species might also have another aspect of habitat preference that is not simply on the eutrophic‐oligotrophic gradient. The group B ponds were negatively characterized with rice paddy cover in the 50‐m buffer zone around the ponds. Rice paddies are habitats for many chironomid species (Darby, 1962; Takamura, 1993), but *P. akamusi* is not included among them. This species might be fairly sensitive to any chemicals like pesticides applied in and around rice paddies.

As shown in Figure 6, the classification of the pond chironomid communities into four groups was not fully conclusive, although it seemed to fit well into this two‐dimensional ordination. Among the classifications of the three, four (Figure 5), and five groups, no identical groups were recognized, but *P. akamusi* was considered as an indicator species in any classification. On the contrary, *T. oyamai*, the indicator species of group C, was regarded as such only in the four‐group classification (Takamura, unpublished). So, *P. akamusi* is strongly representative of a certain type of chironomid community, which may be negatively related to the rice paddy landscape.

One of the important points made in this study is to identify every single specimen of chironomids from quantitative sampling on a molecular basis, even if it did not fully succeed. This type of work is laborious, still possesses methodological limitations in sampling (Shelton et al., 2016), and involved low percentages of successful sequencing (DNA barcoding) at some ponds in this study. In doing so, the specimens, the DNA of which had been sequenced, were delimited to species even though their scientific names were not fully determined due to the shortage of DNA barcode references, difficulties in molecular species delimitation (Tang et al., 2014), or more or less putative delimited species (Zhang et al., 2013). The DNA sequence information collected from these specimens somehow describes the actual states of populations and communities and will be the basis for future species identification or taxonomical reviews. That could provide a deeper understanding of the biodiversity under investigation together with studies on morphological taxonomy and ecology.

The present species delimitation was originally made for the chironomid samples collected from bottom substrates and aquatic plants in the summer and fall of 2012 with the procedures of GMYC: Generalized Mixed Yule Coalescence (Fujisawa & Barraclough, 2013) and PTP: Poisson Tree Process (Zhang et al., 2013) (Takamura et al., 2017). Although their effectiveness has been proven for a variety of data sets, it is clear that they produce slightly different delimitation results for the same data (e.g., Tang et al., 2014). The present results showed the same thing. There were four cases where one GMYC species contained two PTP species and two cases where one contained three (Takamura et al., 2017; Tables S3 and S4, Figures 3 and 4). As chironomid species are hard to identify in their larval stages based on morphological characters (Andersen et al., 2013; Sasa & Kikuch, 1995), it is difficult to determine which delimitation result matches the actual species composition better, based on the comparison between the molecular and morphological identifications. In this study, we selected the result which produced the lowest number of taxa. This decision could be tested by multi‐locus delimitation, thorough morphological identification of larvae backcasted from the present delimitation, and/or identifying adult chironomids reared from future larval collection.

It is worth considering recent advances in species delimitation based on DNA sequences. They have relied more on multiple‐locus data rather than single‐locus data (e.g., Brix et al., 2018; Lin et al., 2017; Luo et al., 2018; Vitecek et al., 2017). Single‐locus data, especially that of the most frequently used gene COI, have a non‐negligible deficiency in delimiting species with gene flow. So, species delimitation based on a larger number of loci is ideal, especially for closely related species (Dupuis et al., 2012; Luo et al., 2018). There were some delimited species that might contain two or more closely related species in this study. For example, *Microchironomus tener* was delimited as a single species by GMYC, but as three species by PTP (Tables S3 and S4, Figures 3 and 4).

The present collection of chironomid larvae came from 17 ponds situated within an area of about 30 km by 30 km. Chironomids are generally not regarded as actively dispersing insects, but they are likely to be easily dispersed on wind and water currents (Armitage, 1995). In this regard, the collection of each species could be supposed to have come from a population or a meta‐population. So, the samples and their phylogenetic relationship may well reflect the coalescence processes supposed to be proceeding in the area. The clump of short branches at the tip of the phylogenetic tree (Figures 3 and 4) can be seen as a species population entity in which the coalescence process has proceeded. This branching structure seems to clearly demonstrate the threshold level where the processes of speciation and coalescence are discriminated from each other. If this were not the case, the discrimination process central to the species delimitation models would not have worked efficiently. This implies that the present collection of chironomids is ideal for molecular species delimitation.

The present results on diversity of chironomid species composition between the farm ponds were observed almost ten years ago. Now the environmental conditions in and around the farm ponds have changed along with the trend stated in Introduction. Although it is the object of future research how chironomid communities have responded to the change, it is noteworthy that the toxic effects of neonicotinoids have been increasingly noticed and investigated not only for terrestrial arthropods like bees, but also for aquatic insects (e.g., Hladik et al., 2018; Morrissey et al., 2015; Takeshita et al., 2020). The association of chironomid community ordination with rice paddy land use may suggest the effect, while no pesticides were monitored in this study. As discussed by Imai et al. (2016) and Usio et al. (2013), Usio et al. (2017), the style of pond management and people's (farmers and nonfarmers) concerns on the activity is also worth considering for the conservation of pond biodiversity. In both points, monitoring the status of biodiversity in ponds is a key in assessing and making a feedback to the conservation. DNA barcoding and molecular species delimitation would make a great contribution.

## CONFLICT OF INTEREST

The authors declare no competing interests.

## AUTHOR CONTRIBUTION

**Kenzi Takamura:** Conceptualization (lead); Data curation (equal); Funding acquisition (lead); Investigation (lead); Methodology (lead); Project administration (lead); Writing‐original draft (lead); Writing‐review & editing (equal). **Ryuhei Ueno:** Resources (lead). **Natsuko Ito Kondo:** Data curation (lead); Methodology (lead); Writing‐review & editing (lead). **Kako Ohbayashi:** Data curation (lead); Methodology (lead).

## Supporting information

Table S1‐S4

## Data Availability

DNA sequences of COI mitochondrial gene of delimited specimens were deposited in DNA Data Bank of Japan (LC494722–LC495141).
