# Supplementary material for: Pond chironomid communities revealed by molecular species delimitation reflect eutrophication
Source: Ecol Evol. 2021 Mar 23;11(9):4193–204. doi: 10.1002/ece3.7315 (PMC8093717; doi:10.1002/ece3.7315)
Supplement: Supplementary file 1 — Table S1‐S4 [file ECE3-11-4193-s001.docx]

| **Table S3**. List of specimen IDs in Figure 3 and corresponding species names. | |
| --- | --- |
| Specimen ID | Species name |
| A0248 | *Tanytarsus mendax* |
| A0563, A0566, A0572 | Sp68 |
| B0275 | Sp53 |
| A0099, A0207, B0180 | *Tanytarsus unagiseptimus* |
| A0357 | *Tanytarsus tamagotoi* |
| A0021 | *Paratanytarsus grimmii* |
| A0595 | Sp08 |
| A0225, B0501 | Sp03 |
| A0286 | Sp02 |
| A0054, A0083, A0100, A0102, A0105, B0242, B0249, B0505 | *Tanytarsus oyamai* |
| A0469 | Sp27 |
| A0751 | Sp37 |
| B0516, B0520 | Sp72 |
| B0175 | Sp59 |
| A0030, A0032, A0034, A0035, A0039, A0053, A0055, A0071, A0073, A0196, A0557, A0578, A0579, B0007 | *Microchironomus tener* |
| B0092, B0103, B0522 | *Dicrotendipes pelochloris* |
| A0092, A0093, A0094, A0111 | *Glyptotendipes tokunagai* |
| A0133, A0134 | *Glyptotendipes cauliginellus* |
| A0179, A0195 | Sp71 |
| A0192, A0287, A0384, A0493 | *Parachironomus monochromus* |
| B0001 | *Nilodorum tainanus* |
| A0173 | *Parachironomus arcuatus* |
| B0518, B0519 | Sp20 |
| A0341, A0342, A0343, A0344, A0345, A0346, A0348, A0351, A0352, A0358, A0376, A0378, A0379, A0392, A0400, A0415, A0750, A0753, B0536 | Sp18 |
| B0511 | Sp06 |
| A0526, A0530 | Sp40 |
| A0221, A0227, A0228, A0229, A0249, A0250, A0252, A0335, A0337, A0338, A0367, A0408, A0432, A0507, B0203, B0252, B0332, B0461 | *Benthalia* sp. |
| A0515, A0516, A0518, B0162, B0221, B0223, B0323, B0480 | *Benthalia dissidens* |
| A0151, A0155, A0156, A0160, A0169, A0266, A0372, A0373, A0403, A0462, A0489 | *Chironomus fujitertius* |
| A0024, A0025, A0060, A0061, A0062, A0191, A0374, A0539, A0541 | *Chironomus plumosus* |
| A0002, A0003, A0004, A0005, A0009, A0011, A0014, A0015, A0091, A0095, A0115, A0142, A0153, A0154, A0161, A0202, A0211, A0253, A0254, A0255, A0270, B0077, B0158, B0159, B0160, B0217, B0272, B0468, B0469, B0470, B0472, B0474, B0517, B0521 | *Chironomus striatipennis* |
| A0007, A0008, A0018, A0019, B0502 | *Chironomus kiiensis* |
| B0082, B0083, B0085, B0347 | *Chironomus circumdatus* |
| A0096, A0219, B0208 | Sp69 |
| A0159 | *Chironomus yoshimatsui* |
| A0101, A0128 | Sp60 |
| A0097, A0201 | Sp58 |
| A0089, A0237, A0381, A0382, A0581 | *Psectrocladius schlienzi* |
| A0144, A0145, A0231, A0290, A0450, A0584, A0596, A0670, A0708 | *Psectrocladius yunoquartus* |
| A0233 | Sp29 |
| A0200, A0402, A0597, A0632, A0644, A0651, A0657, A0678, A0680, A0682, A0701, A0704, A0705, A0713, A0714, A0722, A0723, A0731, A0734, A0740, B0091 | *Cricotopus tricinctus* |
| A0022, A0075, A0130, A0137, A0146, A0148, A0244, A0612, A0618, A0627, A0638, A0640, A0673, A0674, A0695, B0032, B0033 | *Cricotopus sylvestris* |
| A0766 | Sp14 |
| A0110, A0711 | *Corynoneura* sp K1 |
| A0269, A0310 | *Ablabesmyia prorasha* |
| A0205, A0575, A0582, A0583, A0765, B0506, B0539 | *Ablabesmyia* sp K1 |
| A0591, A0603, A0760, A0762 | Sp45 |
| A0586 | Sp23 |
| A0037, A0049, A0050, B0023 | Sp22 |
| A0172, A0186 | Sp64 |
| A0082, A0165, A0281, A0282, A0289, A0564, B0013, B0147, B0149, B0248, B0365, B0481 | *Psectrotanypus* sp. K1 |
| A0174, A0176, A0177, A0178, A0185, A0307 | *Procladius choreus* |
| A0220 | Sp55 |
| A0081, A0085, A0098, A0113, A0206, A0259, A0487, A0602, B0161, B0165, B0228, B0236, B0270, B0413 | *Tanypus kraatzi* |
| A0027, A0028, A0029, A0031, A0033, A0046, A0048, A0063, A0068 | *Tanypus* sp. |
| A0001, A0043, A0044, A0292, A0316, A0317, A0319, A0324, A0325, A0359, A0360, A0554, B0003, B0004, B0119, B0120, B0122, B0130, B0131 | *Propsilocerus akamusi* |
| B0486 | Sp17 |
| A0222, A0223 | Sp16 |
| A0347, A0388, A0395, A0610 | Sp05 |
| A0340, B0515 | Sp26 |
| A0126, A0496 | Sp24 |
| A0272, A0273, A0274, A0275, A0277, A0288, A0303 | Sp49 |
| A0141, A0209, A0210, A0217, A0218, A0256, A0260, A0261, A0262, A0747 | *Polypedilum nubeculosum* |
| A0125, A0449, A0451, A0464, A0466, A0467, A0635, A0642, A0689, A0692, B0038, B0496 | *Polypedilum cultellatum* |
| A0601 | *Sergentia kizakiensis* |
| A0013, A0103, B0086, B0281 | *Polypedilum nubifer* |
| A0135 | Sp62 |
| A0087 | Sp61 |
| B0093, B0095, B0097, B0098, B0102, B0464, B0526 | Sp42 |
| A0199, A0238, A0247, A0690, B0070 | *Endochironomus pekanus* |
| A0543 | Sp25 |
| A0465 | Sp01 |
| A0010, A0012, A0016, A0077, A0078, A0080, A0117, A0118, A0119, A0121, A0468, A0615, A0621, A0625, A0639, A0654, A0663, A0717, A0725, A0730, A0735, A0738, A0742, A0745, A0746, B0040, B0042, B0043, B0044, B0047, B0048, B0052, B0053, B0057, B0063, B0064, B0065, B0067, B0073, B0075, B0078, B0080, B0523 | *Polypedilum tigrinum* |

| **Table S4.** List of specimen IDs in Figure 4 and corresponding species names. | |
| --- | --- |
| Specimen ID | Species name |
| A0465 | Sp01 |
| A0126, A0496 | Sp24 |
| A0543 | Sp25 |
| A0340, B0515 | Sp26 |
| A0199, A0238, A0247, A0690, B0070 | *Endochironomus pekanus* |
| B0093, B0095, B0097, B0098, B0102, B0464, B0526 | Sp42 |
| A0087 | Sp61 |
| A0135 | Sp62 |
| A0222, A0223 | Sp16 |
| B0486 | Sp17 |
| A0601 | *Sergentia kizakiensis* |
| A0125, A0449, A0451, A0464, A0466, A0467, A0635, A0642, A0689, A0692, B0038, B0496 | *Polypedilum cultellatum* |
| A0141, A0209, A0210, A0217, A0218, A0256, A0260, A0261, A0262, A0747 | *Polypedilum nubeculosum* |
| A0272, A0273, A0274, A0275, A0277, A0288, A0303 | Sp49 |
| A0013, A0103, B0086, B0281 | *Polypedilum nubifer* |
| A0001, A0043, A0044, A0292, A0316, A0317, A0319, A0324, A0325, A0359, A0360, A0554, B0003, B0004, B0119, B0120, B0122, B0130, B0131 | *Propsilocerus akamusi* |
| A0022, A0075, A0130, A0137, A0146, A0148, A0244, A0612, A0618, A0627, A0638, A0640, A0673, A0674, A0695, B0032, B0033 | *Cricotopus sylvestris* |
| A0200, A0402, A0597, A0632, A0644, A0651, A0657, A0678, A0680, A0682, A0701, A0704, A0705, A0713, A0714, A0722, A0723, A0731, A0734, A0740, B0091 | *Cricotopus tricinctus* |
| A0233 | Sp29 |
| A0766 | Sp14 |
| A0144, A0145, A0231, A0290, A0450, A0584, A0596, A0670, A0708 | *Psectrocladius yunoquartus* |
| A0089, A0237, A0381, A0382, A0581 | *Psectrocladius schlienzi* |
| A0027, A0028, A0029, A0031, A0033, A0046, A0048, A0063, A0068 | *Tanypus* sp. |
| A0081, A0085, A0098, A0113, A0206, A0259, A0487, A0602, B0161, B0165, B0228, B0236, B0270, B0413 | *Tanypus kraatzi* |
| A0082, A0165, A0281, A0282, A0289, A0564, B0013, B0147, B0149, B0248, B0365, B0481 | *Psectrotanypus* sp. K1 |
| A0172, A0186 | Sp64 |
| A0174, A0176, A0177, A0178, A0185, A0307 | *Procladius choreus* |
| A0220 | Sp55 |
| A0037, A0049, A0050, B0023 | Sp22 |
| A0586 | Sp23 |
| A0591, A0603, A0760, A0762 | Sp45 |
| A0205, A0575, A0582, A0583, A0765, B0506, B0539 | *Ablabesmyia* sp K1 |
| A0269, A0310 | *Ablabesmyia prorasha* |
| A0110, A0711 | *Corynoneura* sp K1 |
| A0751 | Sp37 |
| B0516, B0520 | Sp72 |
| B0511 | Sp06 |
| A0133, A0134 | *Glyptotendipes cauliginellus* |
| A0092, A0093, A0094, A0111 | *Glyptotendipes tokunagai* |
| B0092, B0103, B0522 | *Dicrotendipes pelochloris* |
| A0030, A0032, A0073, A0578, A0034, A0035, A0039, A0053, A0055, A0196, A0579, B0007, A0071, A0557 | *Microchironomus tener* |
| B0175 | Sp59 |
| B0001 | *Nilodorum tainanus* |
| A0192, A0287, A0384, A0493 | *Parachironomus monochromus* |
| A0341, A0342, A0343, A0344, A0345, A0346, A0348, A0351, A0352, A0358, A0376, A0378, A0379, A0392, A0400, A0415, A0750, A0753, B0536 | Sp18 |
| A0173 | *Parachironomus arcuatus* |
| B0518, B0519 | Sp20 |
| A0195, A0179 | Sp71 |
| A0007, A0008, A0018, A0019, B0502 | *Chironomus kiiensis* |
| A0002, A0003, A0004, A0005, A0009, A0011, A0014, A0015, A0091, A0095, A0115, A0142, A0153, A0154, A0161, A0202, A0211, A0253, A0254, A0255, A0270, B0077, B0158, B0159, B0160, B0217, B0272, B0468, B0469, B0470, B0472, B0474, B0517, B0521 | *Chironomus striatipennis* |
| A0219, B0208, A0096 | Sp69 |
| B0082, B0083, B0085, B0347 | *Chironomus circumdatus* |
| A0097, A0201 | Sp58 |
| A0101, A0128 | Sp60 |
| A0159 | *Chironomus yoshimatsui* |
| A0024, A0025, A0060, A0061, A0062, A0191, A0374, A0539, A0541 | *Chironomus plumosus* |
| A0151, A0155, A0156, A0160, A0169, A0266, A0372, A0373, A0403, A0462, A0489 | *Chironomus fujitertius* |
| A0515, A0516, A0518, B0162, B0221, B0223, B0323, B0480 | *Benthalia dissidens* |
| A0221, A0227, A0228, A0229, A0249, A0250, A0252, A0335, A0337, A0338, A0367, A0408, A0432, A0507, B0203, B0252, B0332, B0461 | *Benthalia* sp. |
| A0526, A0530 | Sp40 |
| A0595 | Sp08 |
| A0021 | *Paratanytarsus grimmii* |
| A0357 | *Tanytarsus tamagotoi* |
| A0469 | Sp27 |
| A0054, A0083, A0100, A0102, A0105, B0242, B0249, B0505 | *Tanytarsus oyamai* |
| A0248 | *Tanytarsus mendax* |
| A0566, A0572, A0563 | Sp68 |
| A0286 | Sp02 |
| A0225, B0501 | Sp03 |
| A0099, A0207, B0180 | *Tanytarsus unagiseptimus* |
| B0275 | Sp53 |
| A0010, A0012, A0016, A0077, A0078, A0080, A0117, A0118, A0119, A0121, A0468, A0615, A0621, A0625, A0639, A0654, A0663, A0717, A0725, A0730, A0735, A0738, A0742, A0745, A0746, B0040, B0042, B0043, B0044, B0047, B0048, B0052, B0053, B0057, B0063, B0064, B0065, B0067, B0073, B0075, B0078, B0080, B0523 | *Polypedilum tigrinum* |
| A0347, A0388, A0395, A0610 | Sp05 |
